# Impact of post-traumatic stress symptoms on the health-related quality of life in a cohort study with chronically critically ill patients and their partners: age matters

**DOI:** 10.1186/s13054-019-2321-0

**Published:** 2019-02-08

**Authors:** Gloria-Beatrice Wintermann, Katja Petrowski, Kerstin Weidner, Bernhard Strauß, Jenny Rosendahl

**Affiliations:** 1 Department of Psychotherapy and Psychosomatic Medicine, Medizinische Fakultät Carl Gustav Carus, Technische Universität Dresden, 01307 Dresden, Germany; 2 Institute of Medical Psychology and Medical Sociology, Clinic and Polyclinic for Psychosomatic Medicine and Psychotherapy, University Medical Center of the Johannes Gutenberg University, Mainz, Germany; 3 Institute of Psychosocial Medicine and Psychotherapy, Jena University Hospital, Friedrich-Schiller University, Jena, Germany; 4 Center for Sepsis Control and Care, Jena University Hospital, Friedrich-Schiller University, Jena, Germany

**Keywords:** Chronic critical illness, Intensive care unit (ICU), Post-traumatic stress symptoms, Sepsis, Partners, Post-Intensive Care Syndrome-Family (PICS-F), Actor-Partner-Interdependence Model (APIM), Health-related quality of life (QoL)

## Abstract

**Background:**

Survivors of an acute critical illness with continuing organ dysfunction and uncontrolled inflammatory responses are prone to become chronically critically ill. As mental sequelae, a post-traumatic stress disorder and an associated decrease in the health-related quality of life (QoL) may occur, not only in the patients but also in their partners. Currently, research on long-term mental distress in chronically critically ill patient-partner dyads, using appropriate dyadic analysis strategies (patients and partners being measured and linked on the same variables) and controlling for contextual factors, is lacking.

**Methods:**

The present study investigates the interdependence of post-traumatic stress symptoms (PTSS) and the health-related QoL in *n* = 70 dyads of chronically critically ill patients and their partners, using the Actor-Partner-Interdependence Model (APIM) under consideration of contextual factors (age, gender, length of partnership). The Post-traumatic Stress Scale (PTSS-10) and Euro-Quality of Life (EQ-5D-3L) were applied in both the patients and their partners, within up to 6 months after the transfer from acute care ICU to post-acute ICU.

**Results:**

Clinically relevant post-traumatic stress symptoms were reported by 17.1% of the patients and 18.6% of the partners. Both the chronically critically ill patients and their partners with more severe post-traumatic stress symptoms also showed a decreased health-related QoL. The latter was more pronounced in male partners compared to female partners or female patients. In younger partners (≤ 57 years), higher values of post-traumatic stress symptoms were associated with a decreased QoL in the patients.

**Conclusions:**

Mental health screening and psychotherapeutic treatment options should be offered to both the chronically critically ill patients and their partners. Future research is required to address the special needs of younger patient-partner dyads, following protracted ICU treatment.

**Trial registration:**

German Clinical Trials Register No. DRKS00003386. Registered 13 November 2011

**Electronic supplementary material:**

The online version of this article (10.1186/s13054-019-2321-0) contains supplementary material, which is available to authorized users.

## Introduction

Survivors of an acute critical illness with continuing organ dysfunction and uncontrolled inflammatory responses are prone to become chronically critically ill [1]. Although there is no consensus about the precise definition, chronic critical illness represents a syndrome comprising multiple clinical features. Primary characteristics are prolonged mechanical ventilation (> 72 h) and the need for elective tracheotomy [2, 3]. Minor criteria are neuromuscular weakness, immunodeficiency, endocrinopathy, malnutrition, anasarca, and psychological distress [4–6]. After the discharge from the intensive care unit (ICU), these patients require a continued high level of intensive nursing care and are at an increased risk for ongoing functional impairments, hospital readmissions, and a high mortality rate [4, 7]. The affected patients are exposed to a protracted critical situation which implies a severe emotional stressor for the whole family system (for systematic reviews, see [8, 9]). As a consequence, a cluster of adverse mental sequelae may occur in family members, including depression, anxiety, and acute/post-traumatic stress disorder (PTSD). These have been referred to as Post-intensive Care Syndrome-Family (PICS-F) [8, 9]. According to the Diagnostic and Statistical Manual of Mental Disorders (DSM-V), the mere learning that a relative or satisfying) to 10 (very satisfying) to 10 (satisfying friend was exposed to a trauma is sufficient to pioneer the etiopathogenesis of PTSD [10].

Current findings in family members of chronically critically ill patients reveal that 16% show clinically relevant symptoms of post-traumatic stress within up to 6 months following the ICU stay. Additionally, these family members show a significantly diminished health-related quality of life (QoL) compared to a normative sample [11]. According to the Theory of Dyadic Illness Management [12], the health outcomes of both patients and their partners may influence each other and necessitate the treatment of the dyad as an interdependent team. In this regard, intercorrelations of post-traumatic stress symptoms between patients and their partners have been found in general ICU patients [13] and following severe sepsis [14]. Furthermore, psychological symptoms of patients and their partners or spouses affected not only their individual health-related QoL (actor effects) but also that of the respective other (partner effects) [14, 15]. Thus, studies including patients and partners should treat the patient-partner dyad as interacting and non-independent unit of analysis using appropriate statistical strategies such as the Actor-Partner-Interdependence Model (APIM) [16]. Currently, there is a lack of studies on mental long-term sequelae in chronically critically ill patient-partner dyads using appropriate dyadic analysis strategies. Besides, there is evidence that several contextual factors may represent risk or protective factors on the dyadic appraisal and management behaviors of the chronically critically situation [12]. Within the aftermath of ICU treatment, psychiatric disorders are more common in partners than other kinship relations (e.g., [17]). Furthermore, there is evidence that especially partners of younger patients [18, 19], of patients with sepsis, and female partners are prone to an increased risk for mental distress [14, 20, 21] (for a review, see [22]). However, these findings have not been unequivocally clarified for chronically critically ill patient-partner dyads [14].

Addressing the psychological needs of the family members, particularly the partners, is of clinical relevance, since high levels of post-traumatic stress symptoms may increase the risk for a full syndromal PTSD. In the long-run, the partners might not appropriately fulfill their role as informal care-givers and surrogate decision makers. As a consequence, irrational or uninformed decision-making may occur, leading to prolonged ICU stays and diminishing the rehabilitation outcome in chronically critically ill patients [22, 23]. Therefore, the main aim of the present study was to investigate the dyadic relation between post-traumatic stress and health-related QoL in a homogeneous sample of chronically critically ill patients and their partners, applying a straightforward time schedule of 6 months after being discharged from acute care ICU. As analysis strategy, the APIM approach was used, considering differential effects of the patients’ and partners’ age and gender.

## Materials and methods

### Setting and procedure

The study was registered at the German Clinical Trials Register (No. DRKS00003386) and approved by the local Ethics Committee of the Friedrich-Schiller University, Jena, Germany (No. 3278-10/11). All chronically critically ill patients provided written informed consent. Partners gave informed consent on the telephone.

### Participants and sample size

Eligibility criteria of the chronically critically ill patients were assessed at admission on post-acute ICU at a large rehabilitation hospital, within 4 weeks after transfer from acute care ICU (t1). Patients were consecutively enrolled vis à vis at bedside. The primary inclusion criterion for the participation in the present study was a diagnosis of a Critical Illness Polyneuropathy (CIP; International Classification of Diseases-10th revision, ICD-10: G62.8 0) or Critical Illness Myopathy (CIM; ICD-10: G72.80). Moreover, the following inclusion criteria had to be fulfilled: age between 18 and 72 years, a minimum ICU stay of 6 days, mechanical ventilation of more than 3 days, sufficient German language skills, informed consent, and a negative evaluation of the delirium test, Confusion Assessment Method for the Intensive Care Unit (CAM-ICU) [24, 25]. Patients were excluded from the present study if they could not communicate (neither verbally nor nonverbally), were somnolent, cognitively impaired (e.g., could not appropriately understand and answer our questions), or were screened positive for a delirium.

Patients were contacted again via telephone, 3 (t2) or 6 (t3) months after the transfer from acute care ICU to post-acute ICU. During the telephone contact, they were asked whether they had a partner who would agree to be interviewed about their partnership, post-traumatic stress symptoms, and health-related QoL. Partners were eligible if the patients agreed that the partner was interviewed as well. Furthermore, partners were included if they were at least 18 years of age, gave oral informed consent for study participation via the telephone, showed sufficient German language skills, and could be regarded as being satisfying) to 10 (very satisfying) to 10 (very closely interrelated with the chronically critically ill patient. The latter was defined as sharing a mutual household with the patient or, at least, having daily contact with the patient and being mostly involved in the chronically critically ill patient’s care decisions [26]. The involvement in the patient’s care was not a necessary prerequisite for the study participation of the partners.

The present study was cross sectional, nested within a prospective cohort study which was described elsewhere [27]. A sub-sample of chronically critically ill patients, with data available of their partners, was used [27].

### Measures

We used the Confusion Assessment Method for the Intensive Care Unit (CAM-ICU, [24, 25]) to screen for the presence of a delirium. The level of consciousness was evaluated using the Richmond Agitation Sedation Scale (RASS, range: − 5 no reaction to + 4 very aggressive). A RASS score between − 3 and + 4 was tolerable for study participation. Attention was assessed with the subtask Attention Screening Examination (ASE). In this sub-task, a series of ten letters is read. Patients have to give signal if they heard the letter “A.” Furthermore, the degree of disorganized thinking was judged with four simple yes-no questions (e.g., “Will a stone float on water?”). A positive rating of the two sub-tasks led to the exclusion from the study.

The severity of post-traumatic stress symptoms was assessed using the German version of the Post-traumatic Stress Scale (PTSS-10, [28, 29]) within up to 6 months following the transfer from acute care ICU to post-acute ICU in both the chronically critically ill patients and their partners. The PTSS-10 consists of ten items which are rated according to the occurrence of post-traumatic symptomatology (e.g., sleep disturbance, nightmares, frequent changes in mood) on a 7-point Likert scale (1 = never, 7 = always). A total score is determined by summing up the scores of all items (range 10–70). A score of more than 35 points is considered to be an adequate cutoff for clinically relevant PTSD symptomatology [29]. Internal consistency and test-retest reliability of the PTSS-10 can be regarded as high (Cronbach’s *α* = .92, test-retest reliability *r* = .89) [30]. In the present study, Cronbach’s *α* for post-traumatic symptomatology was .87 for both patients and their partners.

Health-related quality of life (QoL) was assessed with the Euro-Quality of Life questionnaire (EQ-5D-3L; [31]) within up to 6 months following the transfer to post-acute ICU in both the chronically critically ill patients and their partners. The EQ-5D-3L measures the health-related QoL on five dimensions (mobility, self-care, usual activities, pain/discomfort, and anxiety/depression) which are evaluated on three severity levels (no problems, some or moderate problems, extreme problems or unable). A single one-dimensional index value is generated based on a simple sum score (range 0–100) according to Hinz et al. [32]. In the present study, Cronbach’s *α* for health-related QoL was .74 for the patients and .69 for their partners.

Patients’ medical history (e.g., medical comorbidities, length of ICU stay/mechanical ventilation in acute care ICU, site of infection) was assessed via patient records. Furthermore, the Barthel index was evaluated by a trained study nurse. Performance in 11 domains, including activities of daily living (e.g., fecal incontinence, urinary incontinence, help with grooming/toilet use/feeding), were evaluated. Values of the Barthel index range between 0 and 100. A higher value is associated with a better mobility and degree of independence from caregivers. Additionally, the early rehabilitation Barthel index was assessed with respect to seven domains, i.e., intensive care supervision, tracheostomy tube management and supervision, intermittent or continuous mechanical ventilation, confusion, behavioral disturbances, severe impairment of communication, and dysphagia, with a minimum value of − 325 and a maximum value of 0 [33]. Both Barthel scales were summed up, yielding scores between − 325 and 100. Interrater reliability is very high (*r* = .95), test-retest reliability is good as well (*r* = .89) [34].

Partners rated their perceived satisfaction and satisfying) to 10 (very satisfying) to 10 (very closeness of the relationships with the chronically critically ill patient using numerical rating scales, ranging from 1 (not satisfying) to 10 (very satisfying) to 10 (very close/satisfying) to 10 (very satisfying) to 10 (very satisfying) to 10 (very close/satisfying). For an overview of the measures used in the present study see Additional file 1: Table S1.

### Statistical analysis

Continuous sociodemographic and clinical characteristics are presented as means and standard deviations in case of normally distributed data. For non-normal continuous data, medians and interquartile ranges (IQR) are reported. Categorical variables are reported as absolute and relative frequencies. Bivariate correlation analyses were run between patients and their partners with respect to post-traumatic stress symptoms and the health-related QoL, using Kendall’s tau (*τ*) rank correlation coefficients. Wilcoxon’s signed rank tests were used to compare means of outcome variables (health-related QoL/post-traumatic stress symptoms) between patients and partners. For comparisons of the patients’ and partners’ EQ-5D-3L scores with age- and gender-stratified subgroups of the general German population, standardized mean differences (Hedges’ *g*) with 95% confidence interval (CI) were calculated. Spearman’s rank-order correlation analyses were applied in order to evaluate the effect of clinical/dyadic characteristics (days of ICU stay, days of mechanical ventilation/length of partnership) on post-traumatic stress symptoms, and health-related QoL.

To generate an APIM, multilevel modeling was applied [16] using a paired regression technique allowing for the simultaneous analysis of the impact of a person’s post-traumatic stress symptoms on his/her individual health-related QoL (actor effect) and on his/her partner’s health-related QoL (partner effect). Gender was defined as within-dyads covariate, age as mixed covariate, length of partnership, and post-traumatic stress symptom score as mixed continuous predictor variables. *Z*-standardized values were used for all variables of the APIM.

Three age groups were formed based on the 33th and 66th percentile of the patients’ and partners’ age at follow-up (age group 1, ≤ 57 years; age group 2, 57 to 63 years; age group 3, ≥ 63 years). These age groups were analyzed separately with respect to the correlations between the patient’s PTSS-10 score and the patient’s or respective partner’s EQ-5D-3L score using Kendall’s tau rank correlation coefficients. In order to unravel the distinct influence of the patients’ and partners’ age as well as gender, separate APIM analyses were realized for the three age groups and the gender groups.

We applied a significance level of *α* ≤ 0.05 (two-sided). All the analyses were performed using the software Statistical Package for the Social Sciences (SPSS), version 25 (SPSS Inc., Chicago, IL, USA).

## Results

Of the *N* = 207 enrolled chronically critically ill patients, data of *n* = 70 patient-partner dyads could be collected 3 or 6 months following the transfer to post-acute ICU (see flow chart, Additional file 2: Figure S1). The partners of the chronically critically ill patients were assessed with respect to post-traumatic stress and health-related QoL at a median time of 4.8 months (IQR 3.9–6.5) following the ICU discharge. The partners were on average 1 year younger than the patients (median 61.6, IQR 56.1–66.1). 75.7% of the partners were female. The patients stayed a median time of 62.5 days in ICU (IQR 45.5–99.5). The median time of mechanical ventilation was 48.5 days (IQR 28.8–76.0). The dyadic relationships had a median length of 37 years (IQR 26.5–42.5). The partners evaluated their relationships as very satisfying or satisfying) to 10 (very satisfying) to 10 (very close (median 10.0, IQR 8.0/9.0–10.0). For a detailed description see Table 1.

**Table 1:** Descriptive characteristics of the dyads of patients with chronic critical illness and their partners (*n* = 70)

Characteristic	Patients	Life partner/ Spouse	U/ χ^2^/Z	*p*
Age, years, median (IQR)	61.6 (56.1–66.1)	60.6 (53.7–64.5)	− 2.429	.015* (Z)^a^
Gender, *n* (%)				
Male	53 (75.7)	17 (24.3)		
Female	17 (24.3)	53 (75.7)	17.500	< .001*** (*χ*^2^)^b^
Family status, *n* (%)				
Married	63 (90.0)			
Cohabited	7 (10.0)			
Characteristics of relationship, median (IQR)				
Length of partnership (years)^c^		37.0 (26.5–42.5)		
Satisfaction with relationship (1–10)^c^		10.0 (8.0–10.0)		
satisfying) to 10 (very satisfying) to 10 (very closeness of relationship (1–10)		10.0 (9.0–10.0)		
Living together in mutual household, yes/no, *n* (%)^c^		66 (94.3)/3 (4.3)		
Caring for ill patient at the moment, yes/no, *n* (%)		44 (62.9)/26 (37.1)		
Education, *n* (%)^d^				
< 10 years	19 (27.1)			
≥ 10 years	48 (68.6)			
ICU stay, days median (IQR)	62.5 (45.5–99.5)			
Mechanical ventilation, days, median (IQR)	48.5 (28.8–76.0)			
Sepsis, *n* (%)				
No sepsis	27 (38.6)			
Sepsis	22 (31.4)			
Severe sepsis or septic shock	21 (30.0)			
Site of infection, *n* (%)				
Respiratory	33 (47.1)			
Urinary/genitals	7 (10.0)			
Abdominal	7 (10.0)			
Bones/soft tissue	3 (4.3)			
Wound infection	1 (1.4)			
Heart	1 (1.4)			
Multiple	7 (10.0)			
Others^e^	3 (4.3)			
Unknown	1 (1.4)			
Barthel-Index, median (IQR)				
At admission at post-acute ICU	− 195.0 (− 225.0 to − 95.0)			
At discharge from post-acute ICU	− 25.0 (− 80.0–10.0)			
At discharge from rehabilitation	67.5 (20.0–85.0)			
Time following ICU discharge, months, median (IQR)	4.7 (3.8–6.3) Min 3.0, Max 9.2	4.8 (3.9–6.5) Min 3.8, Max 9.2	− 3.325	.001*** (*Z*)^a^

^a^*p* value from Wilcoxon’s signed rank test

^b^*p* value from McNemar test

^c^*n* = 1 missing value

^d^*n* = 3 missing values

^e^*n* = 1 brain, *n* = 2 central venous catheter

The patients who dropped out were more often single or widowed showed a lower level of education and had a significantly higher Barthel index at discharge from the rehabilitation hospital than the patients who were followed up (see Additional file 3: Table S2). Three quarters of the patients had the diagnosis of an acute respiratory insufficiency, and more than one third had a coronary heart disease or diabetes. There were no significant differences between patients who were included or dropped out with respect to medical comorbidities (see Additional file 4: Table S3).

### Post-traumatic stress and health-related QoL in chronically critically ill patients and their partners

The patients and their partners did not significantly differ with respect to post-traumatic stress symptoms as assessed using the PTSS-10 (median, IQR; patients 20, 14–31; partners 19, 14–29.3; *Z* = − .483, *p* = .629). 17.1% (*n* = 12) of the patients and 18.6% (*n* = 13) of the partners were classified as cases with clinically significant post-traumatic stress symptoms within up to 6 months following the discharge from acute care ICU.

Health-related QoL was lower in patients than in their partners (median, IQR; patients 65, 50–80; partners 90, 80–100; *Z* = − 5.974, *p* < .001). Both patients and their partners showed a significantly lower health-related QoL than the general German population (patients: Hedges’ *g* = − 2.098, 95% CI − 2.343; − 1.854; partners: Hedges’ *g* = − .413, 95% CI − .651; − .175). The length of the partnership/days of the ICU stay/days of mechanical ventilation had no impact on post-traumatic stress symptoms or health-related QoL, since no significant correlations could be shown for the patients (− .154 ≥ Spearman’s rho ≤ .005, *p* ≥ .204) or their partners (− .217 ≥ Spearman’s rho ≤ .057, *p* ≥ .074 ) [data available upon request].

### Dyadic perspective

While the patients’ and their partners’ post-traumatic stress was significantly intercorrelated (PTSS-10, *τ* = .236, *p* = .005), no dyadic association between the patients’ and their partners’ health-related QoL was found (EQ-5D-3L, *τ* = .083, *p* = .384).

There was a significant correlation between the post-traumatic stress and the health-related QoL in both the patients and their partners (patients PTSS-10 × EQ-5D-3L, *τ* = − .402, *p* < .001; partners PTSS-10 × EQ-5D-3L, *τ* = − .319, *p* < .001). The patients and their partners with more severe post-traumatic stress symptoms also reported a significantly lower health-related QoL. Regarding the impact of post-traumatic stress symptoms of the patients or their partners on the respective other’s health-related QoL (partner effect), no significant correlations could be shown (PTSS-10 × EQ-5D-3L, patients: *τ* = − .036, *p* = .691; partners: *τ* = − .124, *p* = .159).

**Fig. 1:**
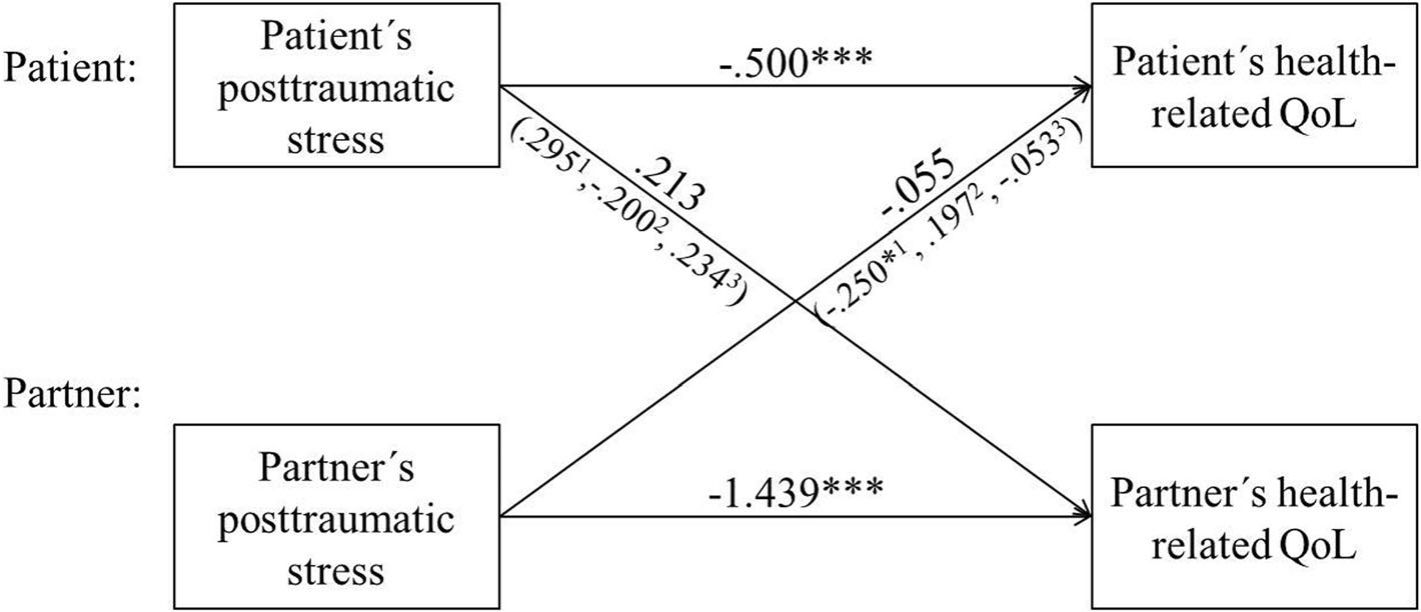
Actor-Partner-Interdependence Model (APIM) testing actor and partner effects of post-traumatic stress symptoms on the health-related quality of life (QoL) in chronically critically ill patients and their partners/spouses. *≤ .05, ***≤ .001; ^1^age group ≤ 57 years, ^2^age group > 57 years< 63 years, ^3^age group ≥ 63 years

The APIM controlling for age and gender, revealed significant actor effects for both the chronically critically ill patients (*β* = − .500, 95% CI − .765 to − .235) and their partners (*β* = − 1.439, 95% CI − 2.138 to − .849) (Fig. 1). No partner effects could be shown for both groups. There was a significant main effect of age and, by trend, for gender. Older age and male gender were associated with a significantly reduced health-related QoL (see Additional file 5: Table S4).

### Impact of age

Significant correlations between a person’s post-traumatic stress symptoms and his/her health-related QoL could be shown for all age groups in the patients and for all but age group 2 in the partners. A significant correlation between the post-traumatic stress symptoms and the respective partner’s health-related QoL was only present in patient-partner dyads aged 57 years or younger (PTSS-10 × EQ-5D-3L, age group 1, partners: *τ* = − .344, *p* = .026) (see Additional file 6: Table S5). More severe post-traumatic stress symptoms in younger partners of chronically critically ill patients were associated with a decreased health-related QoL in the respective patients. This finding could be confirmed using the APIM (*β* = − .250, 95% CI − .493 to − .008) (Table 2, Fig. 1). The impact of the length of the partnership could be ruled out in this model (beta = .139, *T* = 1.218, *p* = .227, CI − .088–.365).

**Table 2:** Actor-Partner-Independence Model (APIM) investigating actor and partner effects of post-traumatic stress symptoms (PTSS-10) and health-related quality of life (EQ-5D-3L) in three different age groups of patients with chronic critical illness and their partners (*n* = 70). Patients and their partners were investigated within up to 6 months after the transfer from acute care ICU to post-acute ICU

Effect	Patients				Partners			
	*β*	95% CI	*t*	*P*	*β*	95% CI	*t*	*P*
PTSS-10 score								
Age group 1 (≤ 57 years)								
Actor effect	− .730	− 1.030, − .430	− 5.062	< .001***	− .705	− .980, − .431	− 5.345	< .001***
Partner effect	.295	− .044, .634	1.810	.085	− .250	− .493, − .008	− 2.145	.044*
− 2 log likelihood	105.258							
Bayes criterion	116.471							
Age group 2 (> 57, < 63 years)								
Actor effect	− .497	− .990, − .004	− 2.108	.049*	− .109	− .662, .443	− .413	.684
Partner effect	− .200	− .641, .240	− .951	.353	.197	− .422, .816	.665	.514
− 2 log likelihood	129.108							
Bayes criterion	140.020							
Age group 3 (≥ 63 years)								
Actor effect	− .307	− .708, .095	− 1.587	− .204	− 1.013	− 1.549, − .477	− 3.928	.001***
Partner effect	.234	− .168, .637	1.213	.239	− .053	− .588, .483	− .204	.840
− 2 log likelihood	113.461							
Bayes criterion	124.674							

Dependent variable: health-related quality of life (EQ-5D-3L, Rabin & de Charro, 2001); *≤ .05, **≤ .01, ***≤ .001

### Impact of gender

The actor × gender interaction reached statistical significance, showing a greater actor effect in male partners than in female partners and female patients (*β* = 1.061, 95% CI .330 to 1.792) (see Additional file 4: Table S3). Testing the APIM separately for men and women did not reveal any significant partner effects (Additional file 7: Table S6).

## Discussion

The primary aim of the present study was to investigate the association between post-traumatic stress and health-related quality of life (QoL) simultaneously in chronically critically ill patients and their partners following the discharge from the intensive care unit (ICU). In summary, our main results revealed that nearly every fifth patient (17.1%) and partner (18.6%) showed clinically relevant symptoms of post-traumatic stress. Additionally, the patients reported a significantly decreased health-related QoL compared to their partners and the general German population. Moreover, more severe post-traumatic stress symptoms were associated with a lower health-related QoL in both the patients and their partners (actor effect). The impact of post-traumatic stress symptoms on the health-related QoL of the respective other (partner effect) was only present in partners of dyads aged 57 years or younger. Finally, male partners showed higher actor effects than female partners and female patients (Additional file 7).

Our rate of clinically relevant post-traumatic stress symptoms is in the range of 13% or 35 to 57% reported by Davidson et al. [8] and van Beusekom et al. [9] for relatives of critically ill patients. With respect to family members or spouses of chronically critically ill patients, there is only one study currently available, which addresses post-traumatic stress symptoms as primary outcome. Rosendahl et al. [14] found 69% and 62%, respectively, of clinically relevant post-traumatic stress symptoms in severe sepsis survivors and their spouses following an average of 55 months after sepsis. However, these heterogeneous rates should be evaluated critically in the context of different samples, assessment methods, and cutoff values used to define the clinical relevance of post-traumatic stress symptoms.

The decreased health-related QoL found in the present study confirms former results (e.g., [9, 14, 35–38]) and adds insight into the kind of special burden chronically critically ill patients, and their partners are suffering from following long-term ICU treatment. These patients belong to a patient cohort requiring prolonged mechanical ventilation, being faced with a high risk for complications during their ICU stay as well as an ongoing high mortality rate, and lasting physical limitations following the ICU discharge [39, 40]. The partners of chronically critically ill patients are often mainly involved in informal caregiving in the aftermath following ICU treatment. In the present study, about two thirds of the partners reported being the caregiver for the chronically critically ill patient at the moment. In line with the present literature, informal care-giving is often associated with decreased physical and emotional health, especially when the patient does not recover fully like in chronically critically ill patients (for a systematic review, see [22, 41, 42]).

In the APIM, significant actor effects in both the patients and their partners were demonstrated, which is in accordance with existing findings [14, 15]. A significant effect of a person’s post-traumatic stress symptomatology on the respective other’s health-related QoL (partner effect) could not be shown, contrasting existing findings in survivors of severe sepsis and patients with heart failure [14, 15]. However, the previously mentioned studies revealed significant partner effects in only one direction. Lacking partner effects in our study might be explained by differences in sample characteristics between studies. In our sample, not only spouses but also life partners were included. Above, Chung et al. [15] only considered spouses who were identified as primary caregivers in patients with heart failure. In our sample, only about two thirds of the partners reported to be involved in the chronically critically ill patient’s care at the moment which may have led to a lack of partner effects. Furthermore, differences between studies according to the measurement of the health-related QoL should be taken into account in order to understand the inconsistency of the findings. While Chung et al. [15] used a disease-specific instrument (the Minnesota Living with Heart Failure Questionnaire), Rosendahl et al. [14] applied the mental component summary of the Short-Form-12 (SF-12) Health Survey. While the latter shows considerable overlap with affective symptoms of post-traumatic stress [43], the EQ-5D-3L used in the present study, only includes one item to evaluate a person’s mental situation (e.g., “I feel extremely anxious or depressed.”). This may have led to a lack of partner effects.

When age groups were formed, particularly younger partners of chronically critically ill patients revealed a significant partner effect on the patients’ health-related QoL. This result confirms findings by Anderson, Arnold, Angus, and Bryce [44] and Gries et al. [45], showing that a younger relative’s or patient’s age displays a major demographic risk factor for the development of the PICS-F. Our finding extends the existing literature from acutely ill patients treated in the ICU to chronically critically ill patients and their partners. Pankrath et al. [46] also showed moderating effects of age in a cohort of chronically ill patients with hematological cancer. Younger patient-partner dyads may be more affected by the chronically critically state of the patients than older patient-partner dyads. The present results suggest that this effect seems to be independent of the length of the partnership. Probably, younger patient-partner dyads (≤ 57 years) showed a greater emotional interdependence due to the major problems evolving from the chronic critical illness during this specific phase of the life span (including, e.g., occupational disability, financial burden, care for minor children). In contrast, older patient-partner dyads are more often adapted to chronic disabilities, and thus, role changes, financial issues, and disruptions of daily activities can be dealt with more effectively than in younger patient-partner dyads [46, 47]. Moreover, younger partners are more often solely involved in the informal caregiving than older partners are. This reinforces satisfying) to 10 (very satisfying) to 10 (very close interactions and emotional transmissions leading to the induction of feelings of empathy in the members of the dyad.

With respect to the impact of gender, our study could not confirm former results for chronically ill patients, showing a transmission of emotional stress only from male patients to their female partners [20]. A larger actor effect of a person’s post-traumatic stress on his/her individual health-related QoL could be shown for male partners compared to female partners and patients. However, this finding should be regarded as rather exploratory, since only about one quarter of our patients were female. However, it may be assumed that men probably perceive the chronic critical illness of their female partners as more profoundly incisive than women, with respect to role changes and disruption of daily routines. Future research is needed, taking into account the perceived satisfaction and satisfying) to 10 (very satisfying) to 10 (very closeness of the partnership as moderating variables. Moreover, future studies should focus on the impact of dyadic coping (e.g., supportive dyadic coping vs. lack of emotional involvement) on the patients’/partners’ outcomes and relationship satisfaction [46].

Although the present study has several strengths such as prospective data assessment, the investigation of a homogeneous sample of chronically critically ill patients and the use of a straightforward time-frame of 6 months, our results should be carefully evaluated in the context of methodological shortcomings. First, we studied a convenience sample of chronically critically ill patients recruited during their weaning in a large rehabilitation hospital. On the whole, this sample is similar to other cohorts recently described in the literature in that the participants and their partners were in their early 60s, most of them were married and had an education of about 10 years [2, 14]. Moreover, our mortality rate is similar to that of existing findings in chronically critically ill patients [2]. Nevertheless, some peculiarities of our present sample need to be pointed out, e.g., males and females were not evenly divided. Beyond that, our patients had stayed in the ICU for about 8 weeks and had been ventilated for about 6 weeks. However, current evidence in chronically critically ill patients is based on patient samples receiving mechanical ventilation/length of ICU stay between 3 and 4 weeks or shorter [2, 14, 36, 48]. Thus, the generalizability of our results is restricted to a highly specialized cohort of patients in need of protracted critical care.

Second, PTSS-10 was used instead of the PTSS-14. The latter presents a more reliable and valid screening instrument for the assessment of post-traumatic stress symptoms since it also represents the diagnostic criteria re-experiencing and numbing [49]. Third, the correlative nature of the present results should be considered since post-traumatic stress and health-related QoL were assessed concurrently. Information regarding physical comorbidities, post-traumatic stress, and health-related QoL before admission to post-acute ICU were not available in both the patients and their partners. Thus, a causal attribution of the present data to, e.g., the protracted treatment on ICU is not possible.

Fourth, the present results should be carefully evaluated in the context of the rather high drop-out rate. Although, our rate of 66.2% mirrors the common clinical situation in chronically critically ill patients [2, 14, 50], more severely ill patients could not be followed up. This may have led to an underestimation of the patients’ and their partners’ psychological distress as well as the partner effects following the ICU treatment. Accordingly, a limited sample sizes of *N* = 70 dyads could have been analyzed. A post hoc power analysis revealed that with 1-*ß* = 72%, our study was underpowered and a minimum sample size of *n* = 84 dyads would have been necessary to reach a power of 80%.

## Conclusions

To our knowledge, this is the first study on the impact of post-traumatic stress symptoms and the health-related QoL in chronically ill patients and their partners following prolonged intensive care. In summary, nearly every fifth chronically critically ill patient and his/her partner suffered from clinically relevant symptoms of post-traumatic stress with a significantly negative impact on the health-related quality of life, especially in male partners. Of utmost importance is the age-dependent partner effect: the severity of post-traumatic stress symptoms of particularly younger partners exerted a significant influence on the patients’ health-related QoL. Further research is required to identify partners of chronically critically ill patients at highest risk for post-traumatic stress [51]. Future studies should therefore allow a profound assessment of the special needs of patient-partner dyads, taking into account the impact of age and gender on the dyadic association between post-traumatic stress and health-related QoL.

## Additional files

Additional file 1: **Table S1.** Overview of the questionnaires and assessment tools applied in the present study. References are provided in the text. (DOCX 13 kb)

Additional file 2: **Figure S1.** Study flow diagram. *n* = 70 chronically critically ill patient-partner dyads were finally analyzed. (JPG 610 kb)

Additional file 3: **Table S2.** Socio-demographic and clinical characteristics of the patients being followed up (*n* = 70) and drop outs (*n* = 137). (DOCX 18 kb)

Additional file 4: **Table S3.** Medical comorbidities of the patients being followed up within up to 6 months after the transfer from acute care ICU to post-acute ICU (*n* = 70) and the dropped out patients (*n* = 137). (DOCX 17 kb)

Additional file 5: **Table S4.** Actor-Partner-Independence Model (APIM) investigating actor and partner effects of post-traumatic stress symptoms (PTSS-10) on the health-related quality of life (EQ-5D-3L) in patients with chronic critical illness and their partners (*n* = 70). Patients and their partners were investigated within up to 6 months after the transfer from acute care ICU to post-acute ICU. (DOCX 16 kb)

Additional file 6: **Table S5.** Correlation between a person’s PTSS-10 score and his/her EQ-5D-3L score or the respective partner’s EQ-5D-3L score in three different age groups of patients with chronic critical illness and their partners. (DOCX 14 kb)

Additional file 7: **Table S6.** Actor-Partner Independence Model (APIM) investigating actor and partner effects of post-traumatic stress symptoms (PTSS-10) and the health-related quality of life (EQ-5D-3L) in male and female patients with chronic critical illness and their partners (*n* = 70). Patients and their partners were investigated within up to 6 months after the transfer from acute care ICU to post-acute ICU. (DOCX 15 kb)
