# Supplementary material for: Impact of post-traumatic stress symptoms on the health-related quality of life in a cohort study with chronically critically ill patients and their partners: age matters
Source: Crit Care. 2019 Feb 8;23:39. doi: 10.1186/s13054-019-2321-0 (PMC6368748; doi:10.1186/s13054-019-2321-0)
Supplement: Supplementary file 1 — Table S1. Overview of the questionnaires and assessment tools applied in the present study. References are provided in the text. (DOCX 13 kb) [file 13054_2019_2321_MOESM1_ESM.docx]

| **Instrument** | **purpose** | **Range/ Cut-off/ syndrome** |
| --- | --- | --- |
| *BI*: Barthel index | an assessment tool to evaluate the severity of illness and need of care | -325 – 100/ no cut-off |
| *CAM-ICU*: Confusion Assessment Method for the Intensive Care Unit | assessment of a delirium | no range/ positive evaluation of the subtasks „Attention Screening Examination“ and „Disorganized thinking“/ risk for delirium |
| *EQ-5D-3L*: Euro-Quality of Life questionnaire | assessment of the health-related quality of life | 0 – 100/ no cut-.off |
| *PTSS-10*: Posttraumatic Stress Scale | assessment of the intensity of posttraumatic stress symptoms | 10 – 70/ > 35/ clinically relevant posttraumatic stress disorder symptoms |
| *RASS*: Richmond Agitation Sedation Scale | assessment of the level of consciousness | -5 – +2/ -3 – +2/ -3 = moderate sedation; +2 = agitated |

Supplementary material

**Table S1:** Overview of the questionnaires and assessment tools applied in the present study. For the references see the text.
