# Supplementary figures and images for: Impact of post-traumatic stress symptoms on the health-related quality of life in a cohort study with chronically critically ill patients and their partners: age matters
Source: Crit Care. 2019 Feb 8;23:39. doi: 10.1186/s13054-019-2321-0 (PMC6368748; doi:10.1186/s13054-019-2321-0)

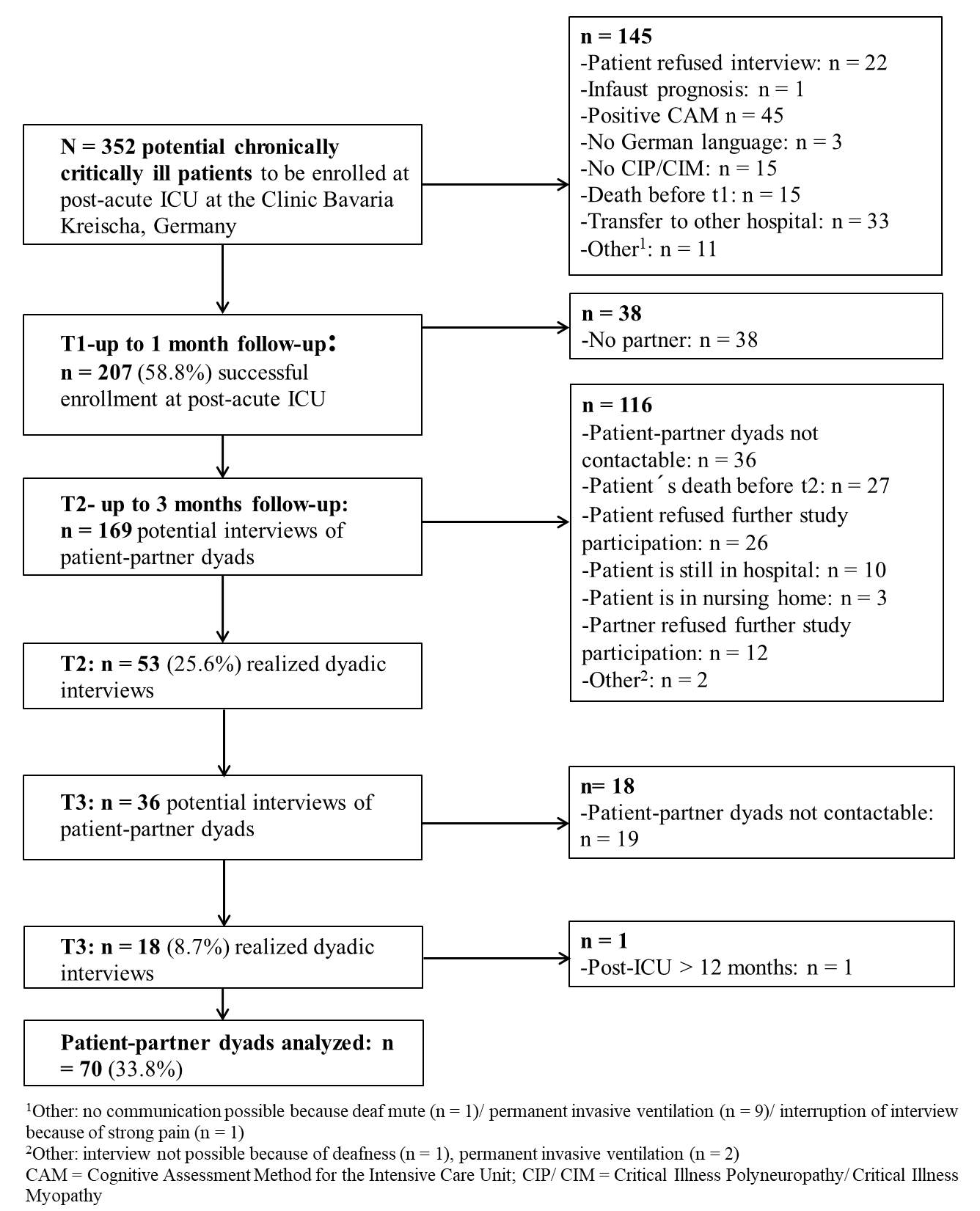

Supplement: Supplementary file 2 — Figure S1. Study flow diagram. n = 70 chronically critically ill patient-partner dyads were finally analyzed. (JPG 610 kb) [file 13054_2019_2321_MOESM2_ESM.jpg]
