# Supplementary material for: Impact of post-traumatic stress symptoms on the health-related quality of life in a cohort study with chronically critically ill patients and their partners: age matters
Source: Crit Care. 2019 Feb 8;23:39. doi: 10.1186/s13054-019-2321-0 (PMC6368748; doi:10.1186/s13054-019-2321-0)
Supplement: Supplementary file 3 — Table S2. Socio-demographic and clinical characteristics of the patients being followed up (n = 70) and drop outs (n = 137). (DOCX 18 kb) [file 13054_2019_2321_MOESM3_ESM.docx]

Supplementary material

**Table S2:** Socio-demographic and clinical characteristics of the patients being followed-up (n = 70) and drop outs (n = 137).

| **Characteristic** | **Patients followed-up**  **n = 70** | **Drop Outs**  **n = 137** | **U/ χ²** | ***p*** |
| --- | --- | --- | --- | --- |
| Age (at time of screening), yrs median (IQR) | 61.3 (55.8-65.9) | 61.5 (55.5-66.0) | 4770.000 | .951 (U)^a^ |
| Gender, n (%) |  |  |  |  |
| Male | 53 (75.7) | 97 (70.8) |  |  |
| Female | 17 (24.3) | 40 (29.2) | .560 | .454 (χ²)^b^ |
| Family status, n (%) |  |  |  |  |
| Married | 63 (90.0) | 72 (52.6) | 43.840 | <.001*** (χ²)^b^ |
| Cohabited | 7 (10.0) | 5 (3.6) |  |  |
| Single |  | 21 (15.3) |  |  |
| Divorced/ living apart |  | 27 (19.7) |  |  |
| Widowed |  | 12 (8.8) |  |  |
| Education, n (%)^c^ |  |  |  |  |
| < 10 yrs | 19 (28.4) | 57 (44.2) |  |  |
| ≥ 10 yrs | 48 (71.6) | 72 (55.8) | 4.653 | .031* (χ²)^b^ |
| ICU stay, days median (IQR) | 62.5 (45.5-99.5) | 69.0 (48.5-98.5) | 4418.500 | .356 (U) |
| Mechanical ventilation , days median (IQR) | 48.5 (28.8-76.0) | 46.0 (32.0-73.0) | 4739.000 | .891 (U) |
| Sepsis, n (%) |  |  |  |  |
| No sepsis | 27 (38.6) | 39 (28.5) |  |  |
| sepsis | 22 (31.4) | 54 (39.4) |  |  |
| Severe sepsis or septic shock | 21 (30.0) | 44 (32.1) | 2.446 | .485 (χ²)^b^ |
| Site of infection, n (%) |  |  |  |  |
| Respiratory | 33 (47.1) | 75 (54.7) | 1.073 | .300 (χ²)^b^ |
| Urinary/ genitals | 7 (10.0) | 12 (8.8) | .086 | .770 (χ²)^b^ |
| Abdominal | 7 (10.0) | 10 (7.3) | .448 | .503 (χ²)^b^ |
| Bones/ soft tissue | 3 (4.3) | 7 (5.1) | .068 | 1.000 (†)^d^ |
| Wound infection | 1 (1.4) | 2 (1.5) | .000 | 1.000 (†)^d^ |
| Heart | 1 (1.4) | 2 (1.5) | .000 | 1.000 (†)^d^ |
| Multiple | 7 (10.0) | 16 (11.7) | .132 | .716 (χ²)^b^ |
| Others^e^ | 3 (4.3) | 15 (10.9) | 2.591 | .107 (†)^d^ |
| Unknown | 1 (1.4) | 6 (4.4) | 1.235 | .266 (†)^d^ |
| Barthel-Index, median (IQR) |  |  |  |  |
| at admission at post-acute ICU | -195.0 (-225.0-  (-)95.0) | -175.0 (-225.0- (-) 112.5) | 4791.000 | .992 (U) |
| at discharge from post-acute ICU | -25.0 (-80.0-11.3) | -40.0 (-100.0- 3.8) | 3986.000 | .056 (U) |
| at discharge from rehabilitation | 67.5 (20.0-85.0) | 50.0 (-22.5-77.5) | 3677.000 | <.006**(U) |

^a^*p*-value from Mann-Whitney U-Test;^b^*p*-value from χ²-test; ^c^ patients followed up: n = 3 missing values; patients dropped out: n = 8 missing values; ^d^*p*-value from Fisher´s exact test; ^e^patients followed up: n = 1 brain, n = 2 central venous catheter; patients dropped out: n = 10 central venous catheter, n = 1 intracardiac catheter, n = 1 nose, n = 2 portsystem, n = 1 aorta; *≤.05, **≤.01, ***≤.001
