# Supplementary material for: Impact of post-traumatic stress symptoms on the health-related quality of life in a cohort study with chronically critically ill patients and their partners: age matters
Source: Crit Care. 2019 Feb 8;23:39. doi: 10.1186/s13054-019-2321-0 (PMC6368748; doi:10.1186/s13054-019-2321-0)
Supplement: Supplementary file 4 — Table S3. Medical comorbidities of the patients being followed up within up to 6 months after the transfer from acute care ICU to post-acute ICU (n = 70) and the dropped out patients (n = 137). (DOCX 17 kb) [file 13054_2019_2321_MOESM4_ESM.docx]

Supplementary material

**Table S3:** Medical comorbidities of the patients being followed-up within up to six months after the transfer from acute care ICU to post-acute ICU (n = 70) and the dropped out patients (n = 137).

| **Characteristic** | **Patients followed-up**  **n = 70** | **Drop outs**  **n = 137** | **χ²** | ***P*** |
| --- | --- | --- | --- | --- |
| **Medical comorbidity** |  |  |  |  |
| **Lung** |  |  |  |  |
| Chronic obstructive pulmonary disease (COPD) (J44.X) | 21 (30.0) | 46 (33.6) | .271 | .603 (χ²)^a^ |
| Acute respiratory insufficiency (J96.00, J96.01, J96.09) | 55 (78.6) | 107 (78.1) | .006 | .938 (χ²)^a^ |
| Chronic respiratory insufficiency (J96.10,  J96.11, J96.19) | 4 (5.7) | 13 (9.5) | .876 | .431 (χ²)^b^ |
| Pneumonia (J15,J18,J69) | 13 (18.6) | 34 (24.8) | 1.030 | .310 (χ²)^a^ |
| Sleep apnea (G47.3) | 6 (8.6) | 15 (10.9) | .287 | .592 (χ²)^a^ |
| **Diseases of the circulatory/ cardiovascular system** |  |  |  |  |
| Left heart failure (I50.1) | 29 (41.4) | 39 (28.5) | 3.528 | .060 (χ²)^a^ |
| Atrial fibrillation (I48.0-I48.2) | 21 (30.0) | 48 (35.0) | .529 | .467 (χ²)^a^ |
| Hypertension (I10.0, I10.01) | 8 (11.4) | 26 (19.0) | 1.924 | .165 (χ²)^a^ |
| Coronary heart disease (I25.1) | 27 (38.6) | 40 (29.2) | 1.860 | .173 (χ²)^a^ |
| **Kidney** |  |  |  |  |
| Chronic kidney disease (N18.X) | 17 (24.3) | 43 (31.4) | 1.135 | .287 (χ²)^a^ |
| Urinary tract infection (N39.0) | 13 (18.6) | 38 (27.7) | 2.096 | .148 (χ²)^a^ |
| **Other** |  |  |  |  |
| Diabetes (E11.90) | 23 (32.9) | 43 (31.4) | .046 | .830 (χ²)^a^ |
| Adipositas (E66.X) | 18 (25.7) | 30 (21.9) | .379 | .538 (χ²)^a^ |
| Enzephalopathy (G93.4) | 15 (21.4) | 36 (26.3) | .587 | .444 (χ²)^a^ |
| Organic brain syndrome (F06.9) | 26 (37.1) | 62 (45.3) | 1.248 | .264 (χ²)^a^ |
| Neurological disorders | 18 (25.7) | 40 (29.2) | .279 | .598 (χ²)^a^ |
| Cirrhosis of the liver | 1 (1.4) | 5 (3.6) | .812 | .666 (†)^b^ |
| Hypothyroidism | 16 (22.9) | 29 (21.2) | .078 | .780 (χ²)^a^ |
| **Mental disorders** |  |  |  |  |
| History of depressive disorders | 13 (18.6) | 36 (26.3) | 1.523 | .217 (χ²)^a^ |
| History of anxiety disorders | 9 (12.9) | 11 (8.0) | 1.237 | .266 (χ²)^a^ |
| History of harmful alcohol consumption | 11 (15.7) | 32 (23.4) | 1.645 | .200 (χ²)^a^ |

^a^*p*-value from χ²-test; ^b^*p*-value from Fisher´s exact test

3
