# Supplementary material for: Impact of post-traumatic stress symptoms on the health-related quality of life in a cohort study with chronically critically ill patients and their partners: age matters
Source: Crit Care. 2019 Feb 8;23:39. doi: 10.1186/s13054-019-2321-0 (PMC6368748; doi:10.1186/s13054-019-2321-0)
Supplement: Supplementary file 5 — Table S4. Actor-Partner-Independence Model (APIM) investigating actor and partner effects of post-traumatic stress symptoms (PTSS-10) on the health-related quality of life (EQ-5D-3L) in patients with chronic critical illness and their partners (n = 70). Patients and their partners were investigated within up to 6 months after the transfer from acute care ICU to post-acute ICU. (DOCX 16 kb) [file 13054_2019_2321_MOESM5_ESM.docx]

Supplementary material

**Table S4:** Actor-Partner-Independence Model (APIM) investigating actor and partner effects of posttraumatic stress symptoms (PTSS-10) on the health-related quality of life (EQ-5D-3L) in patients with chronic critical illness and their partners (N = 70). Patients and their partners were investigated within up to six months after the transfer from acute care ICU to post-acute ICU.

|  | **Patients** | | | | **Partners** | | | |
| --- | --- | --- | --- | --- | --- | --- | --- | --- |
| **Effect** | **β** | **95% CI** | **t** | **P** | **β** | **95% CI** | **t** | **P** |
| **PTSS-10 score** | | | | | | | | |
| Age | -.184 | -.353;-.016 | -2.172 | .033* |  |  |  |  |
| Gender | .373 | -.003;.749 | 1.982 | .052 |  |  |  |  |
|  |  |  |  |  |  |  |  |  |
| Actor effect | -.500 | -.765;-.235 | -3.772 | <.001*** | -1.439 | -2.138;-.849 | -4.621 | <.001*** |
| Partner effect | .213 | -.207; .632 | 1.013 | .315 | -.055 | -.300;.190 | -.450 | .654 |
| Actor x age | -.006 | -.243;.231 | -.050 | .960 | .242 | -.021; .506 | 1.840 | .071 |
| Partner x age | -.040 | -346;.267 | -.259 | .796 | .019 | -.280;.318 | .129 | .898 |
| Actor x gender | .181 | -.299;.661 | .754 | .454 | 1.061 | .330;  1.792 | 2.895 | .005** |
| Partner x gender | -.167 | -692;.359 | -.634 | .528 | .093 | -.601;.788 | .268 | .789 |
|  |  |  |  |  |  |  |  |  |
| -2 log likelihodd | 366.434 |  |  |  |  |  |  |  |
| Bayes Criterion | 380.895 |  |  |  |  |  |  |  |

Dependent variable: health-related quality of life (EQ-5D-3L, Rabin & de Charro, 2001); *≤.05, **≤.01, ***≤.001
