# Supplementary material for: Impact of post-traumatic stress symptoms on the health-related quality of life in a cohort study with chronically critically ill patients and their partners: age matters
Source: Crit Care. 2019 Feb 8;23:39. doi: 10.1186/s13054-019-2321-0 (PMC6368748; doi:10.1186/s13054-019-2321-0)
Supplement: Supplementary file 6 — Table S5. Correlation between a person’s PTSS-10 score and his/her EQ-5D-3L score or the respective partner’s EQ-5D-3L score in three different age groups of patients with chronic critical illness and their partners. (DOCX 14 kb) [file 13054_2019_2321_MOESM6_ESM.docx]

Supplementary material

**Table S5:** Correlation between a person´s PTSS-10 score and his/ her EQ-5D-3L score or the respective partner´s EQ-5D-3L score in three different age groups of patients with chronic critical illness and their partners.

|  | **Age group 1 (<=57 yrs)**  **n = 24** | **Age group 2 (>57 yrs, <63 yrs)**  **n = 22** | **Age group 1 (>=63 yrs)**  **n = 24** |
| --- | --- | --- | --- |
| Patient´s PTSS-10 x patient´s EQ-5D-3L | -.694*** | -.354* | -.318* |
| Partner´s PTSS-10 x partner´s EQ-5D-3L | -.527*** | -.137 | -.366* |
| Patient´s PTSS-10 x Partner´s EQ-5D-3L | -.013 | -.073 | .094 |
| Partner´s PTSS-10 x Patient´s EQ-5D-3L | -.344* | .052 | -.066 |

*≤.05, ***≤.001
