# Supplementary material for: Impact of post-traumatic stress symptoms on the health-related quality of life in a cohort study with chronically critically ill patients and their partners: age matters
Source: Crit Care. 2019 Feb 8;23:39. doi: 10.1186/s13054-019-2321-0 (PMC6368748; doi:10.1186/s13054-019-2321-0)
Supplement: Supplementary file 7 — Table S6. Actor-Partner Independence Model (APIM) investigating actor and partner effects of post-traumatic stress symptoms (PTSS-10) and the health-related quality of life (EQ-5D-3L) in male and female patients with chronic critical illness and their partners (n = 70). Patients and their partners were investigated within up to 6 months after the transfer from acute care ICU to post-acute ICU. (DOCX 15 kb) [file 13054_2019_2321_MOESM7_ESM.docx]

Supplementary material

**Table S6:** Actor-Partner Independence Model (APIM) investigating actor and partner effects of posttraumatic stress symptoms (PTSS-10) and the health-related quality of life (EQ-5D-3L) in male and female patients with chronic critical illness and their partners (N = 70). Patients and their partners were investigated within up to six months after the transfer from acute care ICU to post-acute ICU.

|  | **Patients** | | | | **Partners** | | | |
| --- | --- | --- | --- | --- | --- | --- | --- | --- |
| **Effect** | **β** | **95% CI** | **t** | **P** | **β** | **95% CI** | **t** | **P** |
| **PTSS-10 score** | | | | | | | | |
| **male** | | | | | | | | |
|  |  |  |  |  |  |  |  |  |
| Actor effect | -.500 | -.787;-.213 | -3.503 | .001*** | -1.291 | -.044; .634 | -2.153 | .052 |
| Partner effect | .216 | -.327; .759 | .860 | .406 | -.053 | -.318;.211 | -.404 | .688 |
| -2 log likelihodd | 186.909 |  |  |  |  |  |  |  |
| Bayes Criterion | 199.142 |  |  |  |  |  |  |  |
|  |  |  |  |  |  |  |  |  |
| **female** | | | | | | | | |
|  |  |  |  |  |  |  |  |  |
| Actor effect | -.341 | -.651;-.030 | -2.404 | .034* | -.423 | -.713; -.133 | -2.930 | .005** |
| Partner effect | .041 | -.249; .332 | .287 | .775 | .330 | -.285;.945 | 1.170 | .265 |
| -2 log likelihodd | 176.045 |  |  |  |  |  |  |  |
| Bayes Criterion | 188.278 |  |  |  |  |  |  |  |
|  |  |  |  |  |  |  |  |  |

Dependent variable: health-related quality of life (EQ-5D-3L, Rabin & de Charro, 2001); *≤.05, **≤.01, ***≤.001
